# A Pilot Study on Relationships of the Workplace Intergenerational Climate Scale with Other Ageism and Sexism Scales

**DOI:** 10.1093/geroni/igaa057.3219

**Published:** 2020-12-16

**Authors:** Sangkyung Bae, Moon Choi

**Affiliations:** KAIST, Daejeon, Taejon-jikhalsi, Republic of Korea

## Abstract

Due to the demographic changes such as longevity and low birthrates, the proportion of workers over 55 years old is expected to rise more than twice in the coming years. As the age diversity in the workplace is increasing, ageism needs more attention in every context. This study aims to explore relationships of the workplace intergenerational climate scale with other ageism and sexism scales in workplace and non-workplace contexts. Data came from a pilot online survey conducted in South Korea in December 2019 (N=74; average age=46.8 years old ranged from 20 to 76), and the data was analyzed using a series of ANOVAs and logistic regressions. The results showed that chronological age did not have a linear relationship with ageist attitudes in the workplace although relatively younger groups tend to have more ageist attitudes compared to their counterparts. In particular, those in their thirties were most reluctant to work with different generations. Conspicuously, negative attitudes towards working with different generations in the workplace were statistically significantly related to ageist attitudes towards older adults in non-workplace contexts as well as sexist attitudes in the workplace. The findings imply that prejudice and stereotypes towards different age and gender groups in workplace and non-workplace contexts might be intertwined, and interventions reducing ageism in the workplace might also have positive impacts on alleviating other types of ageism and sexism while promoting diversity.

